# 3,3′-[1,2-Phenyl­enebis(methyl­ene)]bis­(1-propyl­benzimidazolium) dibromide hemihydrate

**DOI:** 10.1107/S1600536812001596

**Published:** 2012-01-21

**Authors:** Muhammad Adnan Iqbal, Rosenani A. Haque, Hoong-Kun Fun, Tze Shyang Chia

**Affiliations:** a School of Chemical Sciences, Universiti Sains Malaysia, 11800 USM, Penang, Malaysia; b X-ray Crystallography Unit, School of Physics, Universiti Sains Malaysia, 11800 USM, Penang, Malaysia

## Abstract

The asymmetric unit of the title compound, C_28_H_32_N_4_^2+^·2Br^−^·0.5H_2_O, contains one 3,3′-[1,2-phenyl­enebis(methyl­ene)]bis­(1-propyl­benzimidazolium) cation, two bromide anions and one half-mol­ecule of water. In the crystal, the whole 3,3′-[1,2-phen­yl­enebis(methyl­ene)]bis­(1-propyl­benzimidazolium) cation and one of the bromide anions are each disordered over two positions with site-occupancy ratios of 0.751 (6):0.249 (6) and 0.680 (8):0.320 (8). For the major component of the disordered cation, the central benzene ring forms dihedral angles of 83.6 (5) and 88.2 (4)° with its adjacent imidazole rings, while these angles for the minor component are 89.2 (15) and 84.9 (13)°. In the crystal, the cations and anions are linked by C—H⋯Br hydrogen bonds into dimers and then stacked along the *c* axis. The crystal packing is further stabilized by C—H⋯π and π–π inter­actions [shortest centroid–centroid distance = 3.646 (4) Å].

## Related literature

For details and applications (biological and catalytic) of *N*-heterocyclic carbenes, see: Herrmann (2002 ▶); Winkelmann & Navarro (2010 ▶); Kascatan-Nebioglu *et al.* (2007 ▶); Ruan *et al.* (2009 ▶); Barnard *et al.* (2004 ▶); Teyssot *et al.* (2009 ▶); Herrmann *et al.* (1995 ▶, 1996 ▶); Cheng & Trudell (2001 ▶); Lee & Hartwig (2001 ▶); Weskamp *et al.* (1998 ▶); Choi *et al.* (2001 ▶). For a related structure, see: Haque *et al.* (2011 ▶).
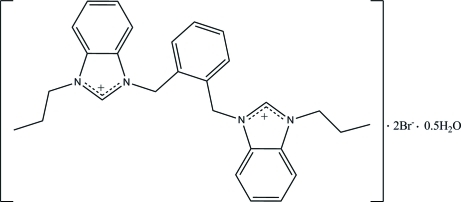


## Experimental

### 

#### Crystal data

C_28_H_32_N_4_^2+^·2Br^−^·0.5H_2_O
*M* *_r_* = 593.40
Triclinic, 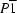

*a* = 9.0465 (4) Å
*b* = 11.4348 (5) Å
*c* = 14.4143 (7) Å
α = 74.013 (1)°
β = 82.935 (1)°
γ = 70.817 (1)°
*V* = 1352.96 (11) Å^3^
*Z* = 2
Mo *K*α radiation
μ = 3.02 mm^−1^
*T* = 296 K
0.54 × 0.44 × 0.26 mm

#### Data collection

Bruker APEX DUO CCD area-detector diffractometer
Absorption correction: multi-scan (*SADABS*; Bruker, 2009 ▶) *T*_min_ = 0.290, *T*_max_ = 0.505
39695 measured reflections
10937 independent reflections
6959 reflections with *I* > 2σ(*I*)
*R*_int_ = 0.033

#### Refinement

*R*[*F* ^2^ > 2σ(*F* ^2^)] = 0.041
*wR*(*F* ^2^) = 0.133
*S* = 1.01
10937 reflections
601 parameters
86 restraints
H-atom parameters constrained
Δρ_max_ = 0.58 e Å^−3^
Δρ_min_ = −0.47 e Å^−3^

### 

Data collection: *APEX2* (Bruker, 2009 ▶); cell refinement: *SAINT* (Bruker, 2009 ▶); data reduction: *SAINT*; program(s) used to solve structure: *SHELXTL* (Sheldrick, 2008 ▶); program(s) used to refine structure: *SHELXTL*; molecular graphics: *SHELXTL*; software used to prepare material for publication: *SHELXTL* and *PLATON* (Spek, 2009 ▶).

## Supplementary Material

Crystal structure: contains datablock(s) global, I. DOI: 10.1107/S1600536812001596/tk5046sup1.cif

Structure factors: contains datablock(s) I. DOI: 10.1107/S1600536812001596/tk5046Isup2.hkl

Supplementary material file. DOI: 10.1107/S1600536812001596/tk5046Isup3.cml

Additional supplementary materials:  crystallographic information; 3D view; checkCIF report

## Figures and Tables

**Table 1:** Hydrogen-bond geometry (Å, °) *Cg*6 and *Cg*8 are the centroids of the N1*X*/C8*X*/C13*X*/N2*X*/C14*X* and C1*X*–C6*X* rings, respectively.

*D*—H⋯*A*	*D*—H	H⋯*A*	*D*⋯*A*	*D*—H⋯*A*
C5—H5*A*⋯Br1^i^	0.93	2.87	3.665 (4)	144
C7—H7*A*⋯Br1^ii^	0.97	2.75	3.596 (9)	146
C14—H14*A*⋯Br1^ii^	0.93	2.87	3.618 (7)	138
C20—H20*A*⋯Br1^ii^	0.93	2.87	3.699 (6)	148
C5—H5*A*⋯*Cg*6	0.93	2.82	3.405 (14)	122
C28—H28*A*⋯*Cg*8^iii^	0.96	2.99	3.607 (15)	123

Symmetry codes: (i) 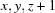; (ii) 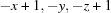; (iii) 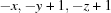.

**Fig. 1.**
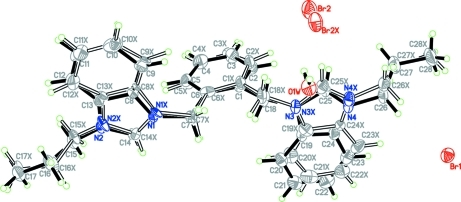
The molecular structure of the title compound with atom labels and 30% probability displacement ellipsoids.

**Fig. 2.**
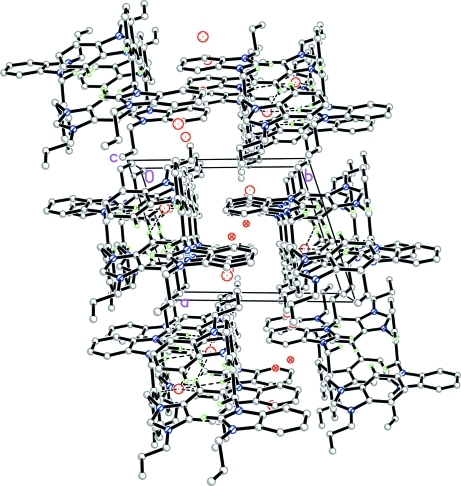
The crystal packing of the title compound. Only the major disordered component is shown. Intermolecular hydrogen bonds are shown as dashed lines and those hydrogen atoms which are not involved in hydrogen bonding are omitted for clarity.

## supplementary crystallographic information

### Comment

*N*-Heterocyclic Carbenes (NHCs) have become universal ligands in organometallic and inorganic coordination chemistry (Herrmann, 2002) as their complexes have widespread biological and catalytic applications (Winkelmann & Navarro, 2010). Metal complexes of NHCs (for example, gold, silver and platinum) have proven to be potential antimicrobial (Kascatan-Nebioglu *et al.*, 2007), antibacterial (Ruan, *et al.*, 2009), antimitochondrial (Barnard *et al.*, 2004) and anticancer (Teyssot *et al.*, 2009) agents. Because of its specific coordination chemistry, this class also exhibits excellent catalytic activity for Heck and Suzuki coupling reactions (Herrmann *et al.*, 1995) as well as aryl amination (Cheng & Trudell, 2001), amide α-arylation (Lee & Hartwig, 2001), hydrosilation (Herrmann *et al.*, 1996), olefin metathesis (Weskamp *et al.*, 1998), and metathesis cross-coupling reactions (Choi *et al.*, 2001).

The molecular structure of the title compound is shown in Fig. 1. The asymmetric unit consists of one 3,3'-[1,2-phenylenebis(methylene)]bis(1-propyl-benzimidazolium) cation, two bromide anions and one half-molecule of water. In the crystal, the whole cation is disordered over two sites with occupancy ratio of 0.751 (6):0.249 (6), whereas the site-occupancy ratio for disordered bromide anion is equal to 0.680 (8):0.320 (8). For the major disordered cation, the central benzene ring (C1–C6) forms dihedral angles of 83.6 (5) and 88.2 (4)°, respectively, with its adjacent imidazole (N1/N2/C8/C13/C14) and (N3/N4/C19/C24/C25) rings; whereas for the minor disordered cation, the central benzene ring (C1X–C6X) forms dihedral angles of 89.2 (15) and 84.9 (13)°, respectively, with its adjacent imidazole (N1X/N2X/C8X/C13X/C14X) and (N3X/N4X/C19X/C24X/C25X) rings. Bond lengths and angles are comparable to related structure (Haque *et al.*, 2011).

In the crystal structure, Fig. 2, the molecules are interconnected by C5—H5A···Br1, C7—H7A···Br1, C14—H14A···Br1 and C20—H20A···Br1 hydrogen bonds (Table 1) into dimers and then stacked along *c* axis. The crystal structure is further stabilized by C—H···π interactions (Table 1). π—π Interactions were also observed with *Cg*_2_···*Cg*_5_ distance = 3.750 (4) Å (symmetry code: 1-*x*, -*y*, 1-*z*), *Cg*_4_···*Cg*_4_ distance = 3.808 (5) Å (symmetry code: 1-*x*, 1-y, 2-*z*) and *Cg*_5_···*Cg*_5_ distance = 3.646 (4) Å (symmetry code: 1-*x*, -*y*, 1-*z*); *Cg*_2_, *Cg*_4_ and *Cg*_5_ are the centroids of N3/N4/C19/C24/C25, C8–C13 and C19–C24 rings, respectively.

### Experimental

A mixture of benzimidazole (2.36 g, 20 mmol) and finely ground potassium hydroxide (2.36 g, 30 mmol) in 30 ml of DMSO was stirred at room temperature (27–28 °C) for 30 minutes. 1-Bromopropane (1.82 ml, 20 mmol) was added drop-wise in this consistently stirred mixture with further stirring for 2 h at the same temperature. The mixture was then poured into water (300 ml) and was extracted by chloroform (5 *x* 20 ml). The extract was dried over magnesium sulfate and evaporated under reduced pressure to get *N*-propylbenzimidazole (1) as a thick yellowish fluid (2.81 g, 88%). Then, a mixture of 1 (1.60 g, 10 mmol) and 1,2-bis(bromomethyl)benzene (1.32 g, 5 mmol) in dioxane (30 ml) was refluxed at 90 °C for 12 h. Desired compound (2.2Br) appeared as white precipitates in the light-brown solution. The mixture was filtered and the precipitates were washed with fresh dioxane (3 *x* 5 ml), dried at room temperature for 24 h, and soft lumps so obtained were ground to fine powder (2.92 g, 63%). Saturated solution of 2.2Br in methanol (0.5 ml) was exposed to diethyl ether vapours at room temperature overnight to get single crystals suitable for X-ray diffraction study.

### Refinement

Atoms H1W1 and H2W1 were located from the difference Fourier map and then fixed at their found location using riding model with *U*_iso_(H) = 1.5*U*_eq_(O) [O—H = 0.8200 and 0.8199 Å]. The remaining H atoms were positioned geometrically [C—H = 0.93, 0.96 or 0.97 Å] and refined using a riding model with *U*_iso_(H) = 1.2 or 1.5*U*_eq_(C). A rotating group model was applied to the methyl group. The 3,3'-[1,2-phenylenebis(methylene)]bis(1-propyl-benzimidazolium) cation and bromine anion are each disordered over two positions with site-occupancy ratios of 0.751 (6):0.249 (6) and 0.680 (8):0.320 (8), respectively. The SAME restraint was employed in the refinement of the disordered components. The same *U_ij_* parameters were used for atoms pairs C1X/C2X, C16X/C17X and C27/C28X. Several outlying reflections, *i.e*. (1 3 2), (12 4), (45 4), (45 3), (56 2) and (1 7 4), were omitted owing to poor agreement. No significant role for the water molecule was found in the crystal structure although a close H1W1···Br2X contact of 2.08 Å is noted with a disordered Br atom.

### Crystal data

**Table:**

C_28_H_32_N_4_^2+^·2Br^−^·0.5H_2_O	*Z* = 2
*M**_r_* = 593.40	*F*(000) = 606
Triclinic, *P*1	*D*_x_ = 1.457 Mg m^−^^3^
Hall symbol: -P 1	Mo *K*α radiation, λ = 0.71073 Å
*a* = 9.0465 (4) Å	Cell parameters from 9923 reflections
*b* = 11.4348 (5) Å	θ = 2.6–29.8°
*c* = 14.4143 (7) Å	µ = 3.02 mm^−^^1^
α = 74.013 (1)°	*T* = 296 K
β = 82.935 (1)°	Block, colourless
γ = 70.817 (1)°	0.54 × 0.44 × 0.26 mm
*V* = 1352.96 (11) Å^3^	

### Data collection

**Table:**

Bruker APEX DUO CCD area-detector diffractometer	10937 independent reflections
Radiation source: fine-focus sealed tube	6959 reflections with *I* > 2σ(*I*)
graphite	*R*_int_ = 0.033
φ and ω scans	θ_max_ = 34.2°, θ_min_ = 2.1°
Absorption correction: multi-scan (*SADABS*; Bruker, 2009)	*h* = −14→14
*T*_min_ = 0.290, *T*_max_ = 0.505	*k* = −17→18
39695 measured reflections	*l* = −22→22

### Refinement

**Table:**

Refinement on *F*^2^	Primary atom site location: structure-invariant direct methods
Least-squares matrix: full	Secondary atom site location: difference Fourier map
*R*[*F*^2^ > 2σ(*F*^2^)] = 0.041	Hydrogen site location: inferred from neighbouring sites
*wR*(*F*^2^) = 0.133	H-atom parameters constrained
*S* = 1.01	*w* = 1/[σ^2^(*F*_o_^2^) + (0.0782*P*)^2^] where *P* = (*F*_o_^2^ + 2*F*_c_^2^)/3
10937 reflections	(Δ/σ)_max_ = 0.001
601 parameters	Δρ_max_ = 0.58 e Å^−^^3^
86 restraints	Δρ_min_ = −0.47 e Å^−^^3^

### Special details

**Table:**

Geometry. All e.s.d.'s (except the e.s.d. in the dihedral angle between two l.s. planes) are estimated using the full covariance matrix. The cell e.s.d.'s are taken into account individually in the estimation of e.s.d.'s in distances, angles and torsion angles; correlations between e.s.d.'s in cell parameters are only used when they are defined by crystal symmetry. An approximate (isotropic) treatment of cell e.s.d.'s is used for estimating e.s.d.'s involving l.s. planes.
Refinement. Refinement of *F*^2^ against ALL reflections. The weighted *R*-factor *wR* and goodness of fit *S* are based on *F*^2^, conventional *R*-factors *R* are based on *F*, with *F* set to zero for negative *F*^2^. The threshold expression of *F*^2^ > σ(*F*^2^) is used only for calculating *R*-factors(gt) *etc*. and is not relevant to the choice of reflections for refinement. *R*-factors based on *F*^2^ are statistically about twice as large as those based on *F*, and *R*- factors based on ALL data will be even larger.

### Fractional atomic coordinates and isotropic or equivalent isotropic displacement parameters (Å^2^)

**Table:**

	*x*	*y*	*z*	*U*_iso_*/*U*_eq_	Occ. (<1)
Br1	0.34759 (2)	0.131872 (19)	0.145379 (13)	0.05001 (8)	
Br2	0.20091 (16)	0.6376 (2)	0.43458 (14)	0.0731 (2)	0.680 (8)
Br2X	0.2357 (6)	0.6020 (5)	0.41783 (18)	0.0640 (8)	0.320 (8)
O1W	0.5457 (4)	0.4487 (4)	0.3898 (2)	0.0707 (9)	0.50
H1W1	0.4629	0.4771	0.4182	0.106*	0.50
H2W1	0.5840	0.5051	0.3609	0.106*	0.50
N1	0.6263 (10)	0.2132 (5)	0.8777 (5)	0.0323 (10)	0.751 (6)
N2	0.7191 (8)	0.1608 (5)	1.0205 (4)	0.0353 (9)	0.751 (6)
N3	0.3066 (7)	0.2152 (6)	0.5574 (4)	0.0406 (11)	0.751 (6)
N4	0.1791 (3)	0.1979 (4)	0.4466 (2)	0.0414 (6)	0.751 (6)
C1	0.3017 (6)	0.2661 (11)	0.7142 (3)	0.0332 (10)	0.751 (6)
C2	0.1394 (6)	0.2895 (9)	0.7207 (4)	0.0468 (15)	0.751 (6)
H2A	0.0857	0.3011	0.6663	0.056*	0.751 (6)
C3	0.0599 (7)	0.2953 (12)	0.8070 (5)	0.062 (2)	0.751 (6)
H3A	−0.0482	0.3129	0.8110	0.074*	0.751 (6)
C4	0.1387 (7)	0.2754 (9)	0.8864 (5)	0.065 (2)	0.751 (6)
H4A	0.0830	0.2801	0.9447	0.078*	0.751 (6)
C5	0.2999 (6)	0.2483 (8)	0.8838 (3)	0.0408 (17)	0.751 (6)
H5A	0.3516	0.2336	0.9398	0.049*	0.751 (6)
C6	0.3839 (6)	0.2434 (8)	0.7963 (3)	0.0291 (14)	0.751 (6)
C7	0.5607 (4)	0.2105 (8)	0.7909 (4)	0.0323 (11)	0.751 (6)
H7A	0.6059	0.1257	0.7798	0.039*	0.751 (6)
H7B	0.5907	0.2705	0.7360	0.039*	0.751 (6)
C8	0.6449 (9)	0.3233 (5)	0.8918 (5)	0.0308 (10)	0.751 (6)
C9	0.6122 (10)	0.4465 (6)	0.8342 (6)	0.0428 (13)	0.751 (6)
H9A	0.5759	0.4681	0.7722	0.051*	0.751 (6)
C10	0.6362 (11)	0.5361 (8)	0.8731 (6)	0.063 (2)	0.751 (6)
H10A	0.6081	0.6212	0.8382	0.075*	0.751 (6)
C11	0.7012 (16)	0.5028 (7)	0.9630 (7)	0.058 (2)	0.751 (6)
H11A	0.7271	0.5637	0.9835	0.070*	0.751 (6)
C12	0.7274 (6)	0.3828 (5)	1.0213 (3)	0.0413 (9)	0.751 (6)
H12A	0.7590	0.3634	1.0842	0.050*	0.751 (6)
C13	0.7053 (12)	0.2882 (6)	0.9837 (5)	0.0355 (14)	0.751 (6)
C14	0.6661 (9)	0.1196 (6)	0.9572 (4)	0.0346 (12)	0.751 (6)
H14A	0.6583	0.0379	0.9673	0.042*	0.751 (6)
C15	0.7712 (8)	0.0843 (10)	1.1177 (5)	0.0462 (15)	0.751 (6)
H15A	0.7519	0.0026	1.1306	0.055*	0.751 (6)
H15B	0.7115	0.1285	1.1654	0.055*	0.751 (6)
C16	0.9454 (6)	0.0619 (7)	1.1263 (4)	0.0479 (13)	0.751 (6)
H16A	1.0063	0.0094	1.0838	0.058*	0.751 (6)
H16B	0.9671	0.1431	1.1071	0.058*	0.751 (6)
C17	0.9910 (10)	−0.0049 (10)	1.2302 (5)	0.0598 (17)	0.751 (6)
H17A	1.1016	−0.0232	1.2352	0.090*	0.751 (6)
H17B	0.9651	−0.0833	1.2497	0.090*	0.751 (6)
H17C	0.9352	0.0498	1.2715	0.090*	0.751 (6)
C18	0.3809 (7)	0.2683 (9)	0.6143 (4)	0.0477 (14)	0.751 (6)
H18A	0.3746	0.3557	0.5803	0.057*	0.751 (6)
H18B	0.4908	0.2188	0.6210	0.057*	0.751 (6)
C19	0.3297 (11)	0.0853 (6)	0.5715 (5)	0.0409 (10)	0.751 (6)
C20	0.4148 (6)	−0.0197 (5)	0.6376 (4)	0.0501 (10)	0.751 (6)
H20A	0.4737	−0.0124	0.6831	0.060*	0.751 (6)
C21	0.4078 (5)	−0.1384 (5)	0.6326 (3)	0.0627 (10)	0.751 (6)
H21A	0.4601	−0.2120	0.6775	0.075*	0.751 (6)
C22	0.3234 (6)	−0.1483 (5)	0.5615 (4)	0.0636 (12)	0.751 (6)
H22A	0.3247	−0.2290	0.5588	0.076*	0.751 (6)
C23	0.2397 (6)	−0.0436 (5)	0.4960 (4)	0.0533 (11)	0.751 (6)
H23A	0.1818	−0.0505	0.4498	0.064*	0.751 (6)
C24	0.2454 (5)	0.0750 (4)	0.5021 (2)	0.0405 (7)	0.751 (6)
C25	0.2186 (5)	0.2804 (4)	0.4814 (3)	0.0419 (7)	0.751 (6)
H25A	0.1893	0.3686	0.4564	0.050*	0.751 (6)
C26	0.0792 (3)	0.2281 (3)	0.36446 (18)	0.0526 (8)	0.751 (6)
H26A	0.1372	0.1801	0.3185	0.063*	0.751 (6)
H26B	−0.0115	0.1994	0.3878	0.063*	0.751 (6)
C27	0.0247 (6)	0.3644 (4)	0.3139 (3)	0.0668 (10)	0.751 (6)
H27A	0.1137	0.3943	0.2883	0.080*	0.751 (6)
H27B	−0.0348	0.4141	0.3585	0.080*	0.751 (6)
C28	−0.0789 (6)	0.3819 (7)	0.2310 (4)	0.0825 (17)	0.751 (6)
H28A	−0.1778	0.4453	0.2366	0.124*	0.751 (6)
H28B	−0.0957	0.3021	0.2344	0.124*	0.751 (6)
H28C	−0.0278	0.4090	0.1703	0.124*	0.751 (6)
N1X	0.616 (3)	0.2328 (16)	0.8647 (14)	0.030 (3)	0.249 (6)
N2X	0.712 (3)	0.1779 (16)	1.0092 (12)	0.048 (4)	0.249 (6)
N3X	0.332 (2)	0.2288 (16)	0.5494 (11)	0.033 (2)	0.249 (6)
N4X	0.2008 (10)	0.2460 (10)	0.4266 (6)	0.0447 (19)	0.249 (6)
C1X	0.289 (2)	0.280 (4)	0.7097 (12)	0.045 (4)	0.249 (6)
C2X	0.131 (2)	0.313 (3)	0.7235 (11)	0.045 (4)	0.249 (6)
H2XA	0.0685	0.3455	0.6698	0.054*	0.249 (6)
C3X	0.0585 (18)	0.302 (3)	0.8143 (10)	0.044 (5)	0.249 (6)
H3XA	−0.0492	0.3162	0.8201	0.053*	0.249 (6)
C4X	0.1405 (12)	0.2703 (18)	0.8960 (10)	0.029 (3)	0.249 (6)
H4XA	0.0912	0.2687	0.9567	0.035*	0.249 (6)
C5X	0.3064 (14)	0.240 (2)	0.8822 (10)	0.048 (6)	0.249 (6)
H5XA	0.3687	0.2137	0.9354	0.058*	0.249 (6)
C6X	0.3755 (16)	0.249 (3)	0.7906 (10)	0.040 (6)	0.249 (6)
C7X	0.5551 (18)	0.214 (3)	0.7805 (14)	0.043 (5)	0.249 (6)
H7XA	0.6001	0.1259	0.7771	0.052*	0.249 (6)
H7XB	0.5847	0.2682	0.7214	0.052*	0.249 (6)
C8X	0.622 (3)	0.3458 (13)	0.8809 (14)	0.025 (2)	0.249 (6)
C9X	0.588 (3)	0.4690 (14)	0.8238 (16)	0.028 (2)	0.249 (6)
H9XA	0.5322	0.4963	0.7675	0.033*	0.249 (6)
C10X	0.644 (2)	0.5502 (16)	0.8580 (13)	0.034 (2)	0.249 (6)
H10B	0.6383	0.6312	0.8192	0.041*	0.249 (6)
C11X	0.706 (5)	0.512 (2)	0.9476 (18)	0.054 (7)	0.249 (6)
H11B	0.7169	0.5735	0.9742	0.065*	0.249 (6)
C12X	0.7528 (18)	0.3874 (17)	0.9997 (9)	0.044 (3)	0.249 (6)
H12B	0.8241	0.3576	1.0486	0.053*	0.249 (6)
C13X	0.683 (3)	0.3077 (16)	0.9728 (15)	0.026 (2)	0.249 (6)
C14X	0.678 (3)	0.1349 (15)	0.9396 (10)	0.026 (2)	0.249 (6)
H14B	0.6953	0.0492	0.9426	0.031*	0.249 (6)
C15X	0.760 (2)	0.106 (3)	1.1083 (13)	0.040 (4)	0.249 (6)
H15C	0.7075	0.0406	1.1293	0.048*	0.249 (6)
H15D	0.7204	0.1639	1.1498	0.048*	0.249 (6)
C16X	0.931 (2)	0.041 (2)	1.1260 (13)	0.049 (3)	0.249 (6)
H16C	0.9888	0.0957	1.0861	0.058*	0.249 (6)
H16D	0.9629	−0.0378	1.1052	0.058*	0.249 (6)
C17X	0.977 (3)	0.009 (3)	1.2318 (13)	0.049 (3)	0.249 (6)
H17D	1.0859	−0.0393	1.2375	0.073*	0.249 (6)
H17E	0.9154	−0.0405	1.2728	0.073*	0.249 (6)
H17F	0.9581	0.0869	1.2509	0.073*	0.249 (6)
C18X	0.385 (2)	0.285 (3)	0.6152 (12)	0.059 (6)	0.249 (6)
H18C	0.3772	0.3729	0.5834	0.071*	0.249 (6)
H18D	0.4941	0.2384	0.6287	0.071*	0.249 (6)
C19X	0.317 (3)	0.1102 (16)	0.5559 (15)	0.048 (5)	0.249 (6)
C20X	0.3762 (18)	−0.0076 (15)	0.6166 (13)	0.062 (4)	0.249 (6)
H20B	0.4266	−0.0160	0.6717	0.074*	0.249 (6)
C21X	0.3613 (18)	−0.1175 (13)	0.5957 (10)	0.069 (4)	0.249 (6)
H21B	0.4040	−0.1983	0.6359	0.082*	0.249 (6)
C22X	0.2820 (16)	−0.1025 (15)	0.5145 (13)	0.061 (4)	0.249 (6)
H22B	0.2701	−0.1742	0.5015	0.073*	0.249 (6)
C23X	0.2211 (12)	0.0149 (13)	0.4533 (11)	0.058 (3)	0.249 (6)
H23B	0.1706	0.0235	0.3983	0.070*	0.249 (6)
C24X	0.2365 (14)	0.1207 (10)	0.4751 (8)	0.037 (2)	0.249 (6)
C25X	0.2511 (14)	0.3105 (12)	0.4735 (9)	0.048 (3)	0.249 (6)
H25B	0.2330	0.3985	0.4561	0.057*	0.249 (6)
C26X	0.1198 (10)	0.3095 (10)	0.3319 (5)	0.053 (2)	0.249 (6)
H26C	0.1340	0.3932	0.3068	0.064*	0.249 (6)
H26D	0.1691	0.2590	0.2859	0.064*	0.249 (6)
C27X	−0.0422 (12)	0.3242 (15)	0.3394 (8)	0.076 (3)	0.249 (6)
H27C	−0.0919	0.3835	0.3791	0.091*	0.249 (6)
H27D	−0.0554	0.2422	0.3738	0.091*	0.249 (6)
C28X	−0.1297 (18)	0.370 (2)	0.2470 (12)	0.076 (3)	0.249 (6)
H28D	−0.2237	0.3462	0.2584	0.114*	0.249 (6)
H28E	−0.0649	0.3326	0.1984	0.114*	0.249 (6)
H28F	−0.1563	0.4618	0.2256	0.114*	0.249 (6)

### Atomic displacement parameters (Å^2^)

**Table:**

	*U*^11^	*U*^22^	*U*^33^	*U*^12^	*U*^13^	*U*^23^
Br1	0.06026 (12)	0.04747 (12)	0.04622 (11)	−0.02155 (9)	−0.00117 (8)	−0.01236 (8)
Br2	0.0514 (3)	0.1019 (5)	0.0664 (4)	−0.0187 (4)	−0.0086 (2)	−0.0250 (5)
Br2X	0.0688 (11)	0.0842 (15)	0.0438 (6)	−0.0383 (10)	−0.0028 (6)	−0.0058 (5)
O1W	0.0696 (19)	0.071 (2)	0.0677 (19)	−0.0031 (17)	−0.0239 (15)	−0.0253 (17)
N1	0.0333 (18)	0.035 (2)	0.031 (2)	−0.0109 (19)	−0.0093 (12)	−0.0079 (19)
N2	0.0299 (16)	0.0447 (17)	0.0320 (16)	−0.0112 (14)	−0.0050 (13)	−0.0093 (15)
N3	0.037 (2)	0.062 (2)	0.0303 (13)	−0.0243 (14)	−0.0014 (14)	−0.0140 (13)
N4	0.0440 (13)	0.056 (2)	0.0321 (11)	−0.0230 (13)	−0.0038 (9)	−0.0141 (13)
C1	0.0260 (12)	0.043 (3)	0.0333 (16)	−0.0110 (13)	−0.0001 (12)	−0.0139 (17)
C2	0.0289 (14)	0.063 (4)	0.054 (2)	−0.0108 (15)	−0.0045 (12)	−0.0249 (19)
C3	0.032 (3)	0.087 (5)	0.075 (4)	−0.015 (3)	−0.004 (2)	−0.035 (3)
C4	0.057 (3)	0.081 (5)	0.055 (3)	−0.017 (3)	0.019 (2)	−0.028 (3)
C5	0.043 (3)	0.054 (3)	0.0253 (16)	−0.015 (2)	0.0067 (15)	−0.0135 (18)
C6	0.031 (2)	0.034 (3)	0.0238 (15)	−0.0086 (17)	−0.0060 (14)	−0.0087 (15)
C7	0.0235 (15)	0.044 (3)	0.0308 (18)	−0.0091 (14)	−0.0001 (12)	−0.0141 (16)
C8	0.023 (3)	0.032 (2)	0.0346 (18)	−0.003 (2)	−0.0052 (13)	−0.0078 (14)
C9	0.038 (3)	0.037 (3)	0.045 (2)	0.000 (2)	−0.0073 (16)	−0.007 (2)
C10	0.069 (3)	0.044 (3)	0.076 (4)	−0.025 (2)	0.014 (2)	−0.014 (2)
C11	0.057 (3)	0.066 (5)	0.072 (4)	−0.026 (4)	0.006 (3)	−0.044 (3)
C12	0.0401 (19)	0.0617 (19)	0.036 (2)	−0.0250 (15)	0.0103 (14)	−0.0278 (16)
C13	0.025 (3)	0.047 (3)	0.0335 (19)	−0.006 (2)	−0.0060 (13)	−0.0138 (19)
C14	0.0310 (17)	0.0408 (19)	0.031 (2)	−0.0164 (14)	−0.0028 (18)	−0.0002 (14)
C15	0.042 (2)	0.060 (3)	0.0313 (18)	−0.0149 (19)	−0.0085 (14)	−0.0015 (18)
C16	0.0298 (13)	0.073 (3)	0.0413 (16)	−0.0172 (17)	−0.0036 (11)	−0.0129 (18)
C17	0.053 (3)	0.069 (3)	0.052 (2)	−0.012 (2)	−0.0166 (17)	−0.008 (2)
C18	0.046 (2)	0.081 (3)	0.036 (2)	−0.037 (2)	0.0074 (16)	−0.027 (2)
C19	0.039 (2)	0.054 (2)	0.0310 (17)	−0.016 (2)	−0.0008 (13)	−0.0121 (18)
C20	0.042 (3)	0.065 (2)	0.0364 (16)	−0.0147 (16)	0.0010 (14)	−0.0046 (13)
C21	0.063 (2)	0.062 (3)	0.047 (2)	−0.0124 (18)	0.0070 (15)	0.0001 (17)
C22	0.070 (3)	0.063 (3)	0.061 (3)	−0.028 (2)	0.017 (2)	−0.018 (2)
C23	0.055 (2)	0.062 (3)	0.050 (2)	−0.025 (2)	0.0036 (18)	−0.019 (2)
C24	0.0387 (12)	0.049 (2)	0.0324 (15)	−0.0130 (17)	−0.0011 (12)	−0.0088 (14)
C25	0.0417 (17)	0.056 (2)	0.0315 (12)	−0.0183 (13)	−0.0068 (11)	−0.0109 (12)
C26	0.0545 (14)	0.0700 (19)	0.0407 (12)	−0.0213 (14)	−0.0154 (10)	−0.0170 (13)
C27	0.080 (3)	0.072 (2)	0.0559 (18)	−0.030 (2)	−0.0285 (18)	−0.0083 (16)
C28	0.098 (4)	0.081 (3)	0.071 (3)	−0.024 (3)	−0.052 (3)	−0.006 (2)
N1X	0.025 (3)	0.026 (4)	0.027 (4)	−0.001 (3)	−0.010 (3)	0.011 (3)
N2X	0.043 (6)	0.059 (8)	0.028 (4)	−0.015 (5)	−0.018 (3)	0.016 (4)
N3X	0.023 (4)	0.055 (5)	0.030 (4)	−0.024 (3)	0.003 (3)	−0.011 (3)
N4X	0.040 (3)	0.061 (6)	0.041 (4)	−0.022 (4)	−0.003 (3)	−0.016 (4)
C1X	0.048 (6)	0.049 (8)	0.040 (5)	−0.015 (5)	−0.016 (4)	−0.008 (4)
C2X	0.048 (6)	0.049 (8)	0.040 (5)	−0.015 (5)	−0.016 (4)	−0.008 (4)
C3X	0.028 (8)	0.061 (10)	0.040 (7)	−0.013 (7)	0.022 (6)	−0.016 (6)
C4X	0.014 (4)	0.054 (8)	0.024 (4)	−0.017 (4)	0.003 (3)	−0.010 (4)
C5X	0.025 (6)	0.050 (9)	0.075 (11)	−0.013 (6)	−0.019 (6)	−0.014 (7)
C6X	0.021 (6)	0.038 (9)	0.066 (11)	−0.015 (5)	0.016 (5)	−0.018 (8)
C7X	0.057 (8)	0.053 (10)	0.031 (5)	−0.020 (6)	−0.022 (4)	−0.017 (5)
C8X	0.016 (5)	0.024 (5)	0.029 (4)	0.008 (4)	−0.014 (3)	−0.010 (4)
C9X	0.026 (5)	0.012 (4)	0.036 (4)	0.005 (4)	−0.015 (3)	0.002 (3)
C10X	0.042 (5)	0.034 (6)	0.036 (4)	−0.021 (4)	−0.002 (4)	−0.011 (4)
C11X	0.077 (15)	0.044 (10)	0.044 (6)	−0.028 (10)	−0.003 (7)	−0.003 (6)
C12X	0.035 (5)	0.076 (8)	0.025 (5)	−0.017 (4)	0.017 (3)	−0.026 (5)
C13X	0.018 (6)	0.022 (4)	0.033 (5)	0.000 (4)	−0.008 (3)	−0.003 (3)
C14X	0.033 (4)	0.032 (5)	0.019 (4)	−0.022 (4)	0.007 (3)	−0.006 (4)
C15X	0.023 (4)	0.061 (9)	0.031 (5)	−0.019 (5)	−0.005 (3)	0.006 (4)
C16X	0.049 (5)	0.071 (7)	0.033 (4)	−0.033 (5)	−0.013 (3)	0.000 (4)
C17X	0.049 (5)	0.071 (7)	0.033 (4)	−0.033 (5)	−0.013 (3)	0.000 (4)
C18X	0.069 (10)	0.071 (10)	0.047 (8)	−0.022 (7)	−0.026 (7)	−0.019 (7)
C19X	0.033 (6)	0.058 (8)	0.045 (8)	−0.011 (6)	0.009 (5)	−0.005 (5)
C20X	0.031 (6)	0.096 (11)	0.059 (10)	−0.023 (6)	0.005 (5)	−0.019 (8)
C21X	0.071 (10)	0.054 (7)	0.050 (8)	−0.006 (7)	0.023 (7)	0.010 (7)
C22X	0.058 (8)	0.061 (10)	0.061 (9)	−0.019 (7)	0.010 (6)	−0.016 (8)
C23X	0.045 (4)	0.071 (8)	0.077 (7)	−0.021 (5)	0.004 (5)	−0.046 (7)
C24X	0.044 (4)	0.031 (5)	0.033 (5)	−0.004 (5)	−0.010 (4)	−0.009 (4)
C25X	0.039 (5)	0.063 (7)	0.053 (5)	−0.022 (4)	−0.001 (4)	−0.025 (5)
C26X	0.051 (4)	0.068 (6)	0.040 (4)	−0.015 (4)	−0.001 (3)	−0.019 (4)
C27X	0.066 (5)	0.090 (8)	0.063 (5)	0.002 (5)	−0.023 (4)	−0.028 (5)
C28X	0.066 (5)	0.090 (8)	0.063 (5)	0.002 (5)	−0.023 (4)	−0.028 (5)

### Geometric parameters (Å, °)

**Table:**

O1W—H1W1	0.8199	C28—H28C	0.9600
O1W—H2W1	0.8200	N1X—C14X	1.343 (13)
N1—C14	1.328 (6)	N1X—C8X	1.393 (13)
N1—C8	1.394 (5)	N1X—C7X	1.484 (13)
N1—C7	1.461 (5)	N2X—C14X	1.337 (13)
N2—C14	1.333 (5)	N2X—C13X	1.377 (13)
N2—C13	1.374 (6)	N2X—C15X	1.477 (13)
N2—C15	1.473 (6)	N3X—C25X	1.337 (13)
N3—C25	1.329 (5)	N3X—C19X	1.385 (13)
N3—C19	1.390 (6)	N3X—C18X	1.478 (14)
N3—C18	1.473 (5)	N4X—C25X	1.334 (11)
N4—C25	1.340 (4)	N4X—C24X	1.359 (11)
N4—C24	1.386 (4)	N4X—C26X	1.510 (10)
N4—C26	1.473 (3)	C1X—C2X	1.362 (13)
C1—C6	1.392 (4)	C1X—C6X	1.382 (13)
C1—C2	1.400 (4)	C1X—C18X	1.520 (14)
C1—C18	1.526 (4)	C2X—C3X	1.382 (13)
C2—C3	1.364 (6)	C2X—H2XA	0.9300
C2—H2A	0.9300	C3X—C4X	1.368 (13)
C3—C4	1.349 (7)	C3X—H3XA	0.9300
C3—H3A	0.9300	C4X—C5X	1.426 (11)
C4—C5	1.386 (6)	C4X—H4XA	0.9300
C4—H4A	0.9300	C5X—C6X	1.382 (13)
C5—C6	1.395 (4)	C5X—H5XA	0.9300
C5—H5A	0.9300	C6X—C7X	1.539 (13)
C6—C7	1.516 (4)	C7X—H7XA	0.9700
C7—H7A	0.9700	C7X—H7XB	0.9700
C7—H7B	0.9700	C8X—C9X	1.377 (13)
C8—C9	1.380 (6)	C8X—C13X	1.399 (13)
C8—C13	1.398 (6)	C9X—C10X	1.408 (12)
C9—C10	1.379 (7)	C9X—H9XA	0.9300
C9—H9A	0.9300	C10X—C11X	1.372 (14)
C10—C11	1.393 (7)	C10X—H10B	0.9300
C10—H10A	0.9300	C11X—C12X	1.362 (15)
C11—C12	1.359 (6)	C11X—H11B	0.9300
C11—H11A	0.9300	C12X—C13X	1.418 (14)
C12—C13	1.413 (5)	C12X—H12B	0.9300
C12—H12A	0.9300	C14X—H14B	0.9300
C14—H14A	0.9300	C15X—C16X	1.497 (15)
C15—C16	1.526 (6)	C15X—H15C	0.9700
C15—H15A	0.9700	C15X—H15D	0.9700
C15—H15B	0.9700	C16X—C17X	1.543 (14)
C16—C17	1.521 (6)	C16X—H16C	0.9700
C16—H16A	0.9700	C16X—H16D	0.9700
C16—H16B	0.9700	C17X—H17D	0.9600
C17—H17A	0.9600	C17X—H17E	0.9600
C17—H17B	0.9600	C17X—H17F	0.9600
C17—H17C	0.9600	C18X—H18C	0.9700
C18—H18A	0.9700	C18X—H18D	0.9700
C18—H18B	0.9700	C19X—C20X	1.364 (14)
C19—C20	1.373 (6)	C19X—C24X	1.403 (13)
C19—C24	1.381 (5)	C20X—C21X	1.420 (15)
C20—C21	1.402 (6)	C20X—H20B	0.9300
C20—H20A	0.9300	C21X—C22X	1.390 (15)
C21—C22	1.400 (6)	C21X—H21B	0.9300
C21—H21A	0.9300	C22X—C23X	1.365 (14)
C22—C23	1.362 (6)	C22X—H22B	0.9300
C22—H22A	0.9300	C23X—C24X	1.384 (11)
C23—C24	1.402 (6)	C23X—H23B	0.9300
C23—H23A	0.9300	C25X—H25B	0.9300
C25—H25A	0.9300	C26X—C27X	1.413 (12)
C26—C27	1.470 (5)	C26X—H26C	0.9700
C26—H26A	0.9700	C26X—H26D	0.9700
C26—H26B	0.9700	C27X—C28X	1.509 (13)
C27—C28	1.538 (4)	C27X—H27C	0.9700
C27—H27A	0.9700	C27X—H27D	0.9700
C27—H27B	0.9700	C28X—H28D	0.9600
C28—H28A	0.9600	C28X—H28E	0.9600
C28—H28B	0.9600	C28X—H28F	0.9600
			
H1W1—O1W—H2W1	111.9	C25X—N3X—C19X	107.1 (11)
C14—N1—C8	108.7 (4)	C25X—N3X—C18X	116.9 (14)
C14—N1—C7	127.8 (5)	C19X—N3X—C18X	134.5 (15)
C8—N1—C7	123.3 (5)	C25X—N4X—C24X	110.4 (8)
C14—N2—C13	109.3 (4)	C25X—N4X—C26X	122.1 (10)
C14—N2—C15	124.9 (6)	C24X—N4X—C26X	127.5 (8)
C13—N2—C15	125.6 (5)	C2X—C1X—C6X	116.5 (12)
C25—N3—C19	109.4 (4)	C2X—C1X—C18X	128.5 (14)
C25—N3—C18	126.4 (5)	C6X—C1X—C18X	114.6 (13)
C19—N3—C18	124.0 (5)	C1X—C2X—C3X	122.5 (14)
C25—N4—C24	108.9 (3)	C1X—C2X—H2XA	118.8
C25—N4—C26	127.4 (3)	C3X—C2X—H2XA	118.8
C24—N4—C26	123.7 (3)	C4X—C3X—C2X	122.0 (13)
C6—C1—C2	120.3 (4)	C4X—C3X—H3XA	119.0
C6—C1—C18	122.8 (4)	C2X—C3X—H3XA	119.0
C2—C1—C18	116.9 (4)	C3X—C4X—C5X	115.8 (11)
C3—C2—C1	120.0 (5)	C3X—C4X—H4XA	122.1
C3—C2—H2A	120.0	C5X—C4X—H4XA	122.1
C1—C2—H2A	120.0	C6X—C5X—C4X	120.7 (11)
C4—C3—C2	119.9 (5)	C6X—C5X—H5XA	119.7
C4—C3—H3A	120.1	C4X—C5X—H5XA	119.7
C2—C3—H3A	120.1	C1X—C6X—C5X	121.9 (11)
C3—C4—C5	122.0 (6)	C1X—C6X—C7X	119.8 (12)
C3—C4—H4A	119.0	C5X—C6X—C7X	118.2 (12)
C5—C4—H4A	119.0	N1X—C7X—C6X	109.7 (14)
C4—C5—C6	119.4 (4)	N1X—C7X—H7XA	109.7
C4—C5—H5A	120.3	C6X—C7X—H7XA	109.7
C6—C5—H5A	120.3	N1X—C7X—H7XB	109.7
C1—C6—C5	118.4 (4)	C6X—C7X—H7XB	109.7
C1—C6—C7	120.6 (4)	H7XA—C7X—H7XB	108.2
C5—C6—C7	121.0 (4)	C9X—C8X—N1X	132.2 (13)
N1—C7—C6	113.7 (4)	C9X—C8X—C13X	123.7 (12)
N1—C7—H7A	108.8	N1X—C8X—C13X	104.0 (11)
C6—C7—H7A	108.8	C8X—C9X—C10X	114.3 (13)
N1—C7—H7B	108.8	C8X—C9X—H9XA	122.8
C6—C7—H7B	108.8	C10X—C9X—H9XA	122.8
H7A—C7—H7B	107.7	C11X—C10X—C9X	121.0 (15)
C9—C8—N1	131.6 (5)	C11X—C10X—H10B	119.5
C9—C8—C13	122.4 (5)	C9X—C10X—H10B	119.5
N1—C8—C13	106.0 (4)	C12X—C11X—C10X	123.7 (17)
C10—C9—C8	116.7 (6)	C12X—C11X—H11B	118.2
C10—C9—H9A	121.7	C10X—C11X—H11B	118.2
C8—C9—H9A	121.7	C11X—C12X—C13X	114.0 (15)
C9—C10—C11	122.0 (6)	C11X—C12X—H12B	123.0
C9—C10—H10A	119.0	C13X—C12X—H12B	123.0
C11—C10—H10A	119.0	N2X—C13X—C8X	109.8 (11)
C12—C11—C10	121.0 (5)	N2X—C13X—C12X	127.4 (15)
C12—C11—H11A	119.5	C8X—C13X—C12X	118.9 (13)
C10—C11—H11A	119.5	N2X—C14X—N1X	110.5 (12)
C11—C12—C13	118.2 (5)	N2X—C14X—H14B	124.7
C11—C12—H12A	120.9	N1X—C14X—H14B	124.7
C13—C12—H12A	120.9	N2X—C15X—C16X	118.6 (16)
N2—C13—C8	106.4 (4)	N2X—C15X—H15C	107.7
N2—C13—C12	134.2 (5)	C16X—C15X—H15C	107.7
C8—C13—C12	119.2 (5)	N2X—C15X—H15D	107.7
N1—C14—N2	109.4 (5)	C16X—C15X—H15D	107.7
N1—C14—H14A	125.3	H15C—C15X—H15D	107.1
N2—C14—H14A	125.3	C15X—C16X—C17X	115.2 (16)
N2—C15—C16	110.8 (6)	C15X—C16X—H16C	108.5
N2—C15—H15A	109.5	C17X—C16X—H16C	108.5
C16—C15—H15A	109.5	C15X—C16X—H16D	108.5
N2—C15—H15B	109.5	C17X—C16X—H16D	108.5
C16—C15—H15B	109.5	H16C—C16X—H16D	107.5
H15A—C15—H15B	108.1	C16X—C17X—H17D	109.5
C17—C16—C15	109.3 (5)	C16X—C17X—H17E	109.5
C17—C16—H16A	109.8	H17D—C17X—H17E	109.5
C15—C16—H16A	109.8	C16X—C17X—H17F	109.5
C17—C16—H16B	109.8	H17D—C17X—H17F	109.5
C15—C16—H16B	109.8	H17E—C17X—H17F	109.5
H16A—C16—H16B	108.3	N3X—C18X—C1X	112.2 (16)
N3—C18—C1	111.4 (5)	N3X—C18X—H18C	109.2
N3—C18—H18A	109.4	C1X—C18X—H18C	109.2
C1—C18—H18A	109.4	N3X—C18X—H18D	109.2
N3—C18—H18B	109.4	C1X—C18X—H18D	109.2
C1—C18—H18B	109.4	H18C—C18X—H18D	107.9
H18A—C18—H18B	108.0	C20X—C19X—N3X	132.9 (13)
C20—C19—C24	122.3 (4)	C20X—C19X—C24X	118.9 (12)
C20—C19—N3	131.5 (4)	N3X—C19X—C24X	107.8 (11)
C24—C19—N3	106.2 (5)	C19X—C20X—C21X	120.0 (13)
C19—C20—C21	116.1 (4)	C19X—C20X—H20B	120.0
C19—C20—H20A	122.0	C21X—C20X—H20B	120.0
C21—C20—H20A	122.0	C22X—C21X—C20X	119.1 (12)
C22—C21—C20	121.3 (4)	C22X—C21X—H21B	120.4
C22—C21—H21A	119.3	C20X—C21X—H21B	120.4
C20—C21—H21A	119.3	C23X—C22X—C21X	121.5 (12)
C23—C22—C21	122.1 (4)	C23X—C22X—H22B	119.2
C23—C22—H22A	118.9	C21X—C22X—H22B	119.2
C21—C22—H22A	118.9	C22X—C23X—C24X	118.4 (11)
C22—C23—C24	116.3 (4)	C22X—C23X—H23B	120.8
C22—C23—H23A	121.8	C24X—C23X—H23B	120.8
C24—C23—H23A	121.8	N4X—C24X—C23X	132.6 (10)
C19—C24—N4	106.8 (4)	N4X—C24X—C19X	105.0 (9)
C19—C24—C23	121.8 (4)	C23X—C24X—C19X	122.0 (11)
N4—C24—C23	131.4 (3)	N4X—C25X—N3X	109.3 (11)
N3—C25—N4	108.7 (4)	N4X—C25X—H25B	125.3
N3—C25—H25A	125.7	N3X—C25X—H25B	125.3
N4—C25—H25A	125.7	C27X—C26X—N4X	113.5 (8)
C27—C26—N4	114.6 (3)	C27X—C26X—H26C	108.9
C27—C26—H26A	108.6	N4X—C26X—H26C	108.9
N4—C26—H26A	108.6	C27X—C26X—H26D	108.9
C27—C26—H26B	108.6	N4X—C26X—H26D	108.9
N4—C26—H26B	108.6	H26C—C26X—H26D	107.7
H26A—C26—H26B	107.6	C26X—C27X—C28X	117.7 (11)
C26—C27—C28	109.0 (3)	C26X—C27X—H27C	107.9
C26—C27—H27A	109.9	C28X—C27X—H27C	107.9
C28—C27—H27A	109.9	C26X—C27X—H27D	107.9
C26—C27—H27B	109.9	C28X—C27X—H27D	107.9
C28—C27—H27B	109.9	H27C—C27X—H27D	107.2
H27A—C27—H27B	108.3	C27X—C28X—H28D	109.5
C14X—N1X—C8X	109.0 (11)	C27X—C28X—H28E	109.5
C14X—N1X—C7X	122.3 (15)	H28D—C28X—H28E	109.5
C8X—N1X—C7X	128.7 (15)	C27X—C28X—H28F	109.5
C14X—N2X—C13X	106.3 (12)	H28D—C28X—H28F	109.5
C14X—N2X—C15X	128.1 (16)	H28E—C28X—H28F	109.5
C13X—N2X—C15X	125.4 (16)		
			
C6—C1—C2—C3	2.7 (12)	C6X—C1X—C2X—C3X	−8(4)
C18—C1—C2—C3	−176.8 (11)	C18X—C1X—C2X—C3X	179 (4)
C1—C2—C3—C4	−1.6 (16)	C1X—C2X—C3X—C4X	8(4)
C2—C3—C4—C5	−0.3 (18)	C2X—C3X—C4X—C5X	−5(4)
C3—C4—C5—C6	1.1 (15)	C3X—C4X—C5X—C6X	3(3)
C2—C1—C6—C5	−1.9 (12)	C2X—C1X—C6X—C5X	6(4)
C18—C1—C6—C5	177.5 (9)	C18X—C1X—C6X—C5X	−180 (3)
C2—C1—C6—C7	176.4 (7)	C2X—C1X—C6X—C7X	−178 (2)
C18—C1—C6—C7	−4.1 (14)	C18X—C1X—C6X—C7X	−4(4)
C4—C5—C6—C1	0.0 (13)	C4X—C5X—C6X—C1X	−4(4)
C4—C5—C6—C7	−178.3 (8)	C4X—C5X—C6X—C7X	−180 (2)
C14—N1—C7—C6	86.5 (11)	C14X—N1X—C7X—C6X	100 (3)
C8—N1—C7—C6	−88.0 (10)	C8X—N1X—C7X—C6X	−80 (3)
C1—C6—C7—N1	169.1 (8)	C1X—C6X—C7X—N1X	159 (3)
C5—C6—C7—N1	−12.7 (11)	C5X—C6X—C7X—N1X	−25 (3)
C14—N1—C8—C9	−176.2 (10)	C14X—N1X—C8X—C9X	173 (3)
C7—N1—C8—C9	−0.7 (15)	C7X—N1X—C8X—C9X	−7(5)
C14—N1—C8—C13	2.6 (10)	C14X—N1X—C8X—C13X	−3(3)
C7—N1—C8—C13	178.0 (8)	C7X—N1X—C8X—C13X	177 (3)
N1—C8—C9—C10	176.1 (10)	N1X—C8X—C9X—C10X	−165 (3)
C13—C8—C9—C10	−2.5 (14)	C13X—C8X—C9X—C10X	10 (4)
C8—C9—C10—C11	4.7 (16)	C8X—C9X—C10X—C11X	−9(4)
C9—C10—C11—C12	−7.6 (19)	C9X—C10X—C11X—C12X	16 (6)
C10—C11—C12—C13	7.7 (16)	C10X—C11X—C12X—C13X	−22 (5)
C14—N2—C13—C8	−1.9 (10)	C14X—N2X—C13X—C8X	4(3)
C15—N2—C13—C8	−177.0 (7)	C15X—N2X—C13X—C8X	−173 (2)
C14—N2—C13—C12	172.6 (10)	C14X—N2X—C13X—C12X	−154 (3)
C15—N2—C13—C12	−2.5 (16)	C15X—N2X—C13X—C12X	30 (5)
C9—C8—C13—N2	178.5 (9)	C9X—C8X—C13X—N2X	−177 (3)
N1—C8—C13—N2	−0.4 (10)	N1X—C8X—C13X—N2X	0(3)
C9—C8—C13—C12	3.0 (14)	C9X—C8X—C13X—C12X	−17 (4)
N1—C8—C13—C12	−175.9 (8)	N1X—C8X—C13X—C12X	159 (2)
C11—C12—C13—N2	−179.4 (11)	C11X—C12X—C13X—N2X	177 (3)
C11—C12—C13—C8	−5.5 (13)	C11X—C12X—C13X—C8X	22 (4)
C8—N1—C14—N2	−3.8 (10)	C13X—N2X—C14X—N1X	−6(3)
C7—N1—C14—N2	−179.0 (7)	C15X—N2X—C14X—N1X	170 (2)
C13—N2—C14—N1	3.6 (10)	C8X—N1X—C14X—N2X	6(3)
C15—N2—C14—N1	178.7 (7)	C7X—N1X—C14X—N2X	−175 (2)
C14—N2—C15—C16	115.6 (9)	C14X—N2X—C15X—C16X	89 (3)
C13—N2—C15—C16	−70.0 (11)	C13X—N2X—C15X—C16X	−96 (4)
N2—C15—C16—C17	174.0 (8)	N2X—C15X—C16X—C17X	159 (2)
C25—N3—C18—C1	109.2 (8)	C25X—N3X—C18X—C1X	106 (2)
C19—N3—C18—C1	−76.3 (10)	C19X—N3X—C18X—C1X	−58 (4)
C6—C1—C18—N3	144.6 (9)	C2X—C1X—C18X—N3X	−47 (4)
C2—C1—C18—N3	−36.0 (10)	C6X—C1X—C18X—N3X	140 (3)
C25—N3—C19—C20	177.8 (9)	C25X—N3X—C19X—C20X	178 (3)
C18—N3—C19—C20	2.4 (15)	C18X—N3X—C19X—C20X	−17 (5)
C25—N3—C19—C24	−2.4 (9)	C25X—N3X—C19X—C24X	6(3)
C18—N3—C19—C24	−177.7 (5)	C18X—N3X—C19X—C24X	171 (2)
C24—C19—C20—C21	−1.8 (12)	N3X—C19X—C20X—C21X	−169 (3)
N3—C19—C20—C21	178.0 (9)	C24X—C19X—C20X—C21X	2(3)
C19—C20—C21—C22	2.5 (9)	C19X—C20X—C21X—C22X	−2(3)
C20—C21—C22—C23	−2.5 (8)	C20X—C21X—C22X—C23X	1(2)
C21—C22—C23—C24	1.5 (7)	C21X—C22X—C23X—C24X	−1.6 (19)
C20—C19—C24—N4	−178.1 (7)	C25X—N4X—C24X—C23X	−174.1 (13)
N3—C19—C24—N4	2.1 (8)	C26X—N4X—C24X—C23X	5(2)
C20—C19—C24—C23	1.0 (12)	C25X—N4X—C24X—C19X	−1.1 (18)
N3—C19—C24—C23	−178.8 (5)	C26X—N4X—C24X—C19X	177.5 (15)
C25—N4—C24—C19	−1.1 (6)	C22X—C23X—C24X—N4X	174.1 (13)
C26—N4—C24—C19	−179.9 (5)	C22X—C23X—C24X—C19X	2(2)
C25—N4—C24—C23	180.0 (4)	C20X—C19X—C24X—N4X	−176 (2)
C26—N4—C24—C23	1.2 (6)	N3X—C19X—C24X—N4X	−3(2)
C22—C23—C24—C19	−0.8 (8)	C20X—C19X—C24X—C23X	−2(3)
C22—C23—C24—N4	178.0 (4)	N3X—C19X—C24X—C23X	171.1 (16)
C19—N3—C25—N4	1.8 (7)	C24X—N4X—C25X—N3X	4.9 (15)
C18—N3—C25—N4	177.0 (5)	C26X—N4X—C25X—N3X	−173.8 (11)
C24—N4—C25—N3	−0.4 (5)	C19X—N3X—C25X—N4X	−7(2)
C26—N4—C25—N3	178.3 (4)	C18X—N3X—C25X—N4X	−174.8 (14)
C25—N4—C26—C27	3.0 (5)	C25X—N4X—C26X—C27X	−108.4 (13)
C24—N4—C26—C27	−178.4 (4)	C24X—N4X—C26X—C27X	73.1 (14)
N4—C26—C27—C28	−179.5 (3)	N4X—C26X—C27X—C28X	−172.1 (13)

### Hydrogen-bond geometry (Å, °)

**Table:**

Cg6 and Cg8 are the centroids of the N1*X*/C8*X*/C13*X*/N2*X*/C14*X* and C1*X*–C6*X* rings, respectively.

**Table:**

*D*—H···*A*	*D*—H	H···*A*	*D*···*A*	*D*—H···*A*
C5—H5A···Br1^i^	0.93	2.87	3.665 (4)	144
C7—H7A···Br1^ii^	0.97	2.75	3.596 (9)	146
C14—H14A···Br1^ii^	0.93	2.87	3.618 (7)	138
C20—H20A···Br1^ii^	0.93	2.87	3.699 (6)	148
C5—H5A···Cg6	0.93	2.82	3.405 (14)	122
C28—H28A···Cg8^iii^	0.96	2.99	3.607 (15)	123

Symmetry codes: (i) *x*, *y*, *z*+1; (ii) −*x*+1, −*y*, −*z*+1; (iii) −*x*, −*y*+1, −*z*+1.
